# The Impact of the Choice of Data Source in Record Linkage Studies Estimating Mortality in Venous Thromboembolism

**DOI:** 10.1371/journal.pone.0148349

**Published:** 2016-02-10

**Authors:** Arlene M. Gallagher, Tim Williams, Hubert G. M. Leufkens, Frank de Vries

**Affiliations:** 1 Clinical Practice Research Datalink, Medicines and Healthcare products Regulatory Agency, London, United Kingdom; 2 Utrecht Institute for Pharmaceutical Sciences, Utrecht University, Utrecht, the Netherlands; Medical University Innsbruck, AUSTRIA

## Abstract

Linked electronic healthcare databases are increasingly being used in observational research. The objective of this study was to investigate the impact of the choice of data source in estimating mortality following VTE, with a secondary aim to investigate the influence of the denominator definition. We used the UK Clinical Practice Research Datalink (CPRD) to identify patients aged 18+ with venous thromboembolism (VTE). Multiple cohorts were identified in order to assess how mortality rates differed with a range of data sources. For each of the cohorts, incidence rates per 1,000 person years (/1000py) and relative rates (RRs) of all-cause mortality were calculated. The lowest mortality rate was found when only primary care data were used for both the exposure (VTE) and the outcome (death) (108.4/1000py). The highest mortality rate was found for patients diagnosed in secondary care (237.2/1000py). When linked primary and secondary care data were included for eligible patients and for the overlapping period of data collection, a mortality rate of 173.2/1000py was found. Sensitivity analyses varying the denominator definition provided a range of results (140.6–164.3/1000py). The relative rates of mortality by gender and age were comparable across all cohorts. Depending on the choice of data source, the population studied may be different. This may have substantial impact on the main findings, in particular on incidence rates of mortality following VTE.

## Introduction

Electronic healthcare data bases are increasingly being used in observational research. For conditions diagnosed, treated and managed solely in primary care, electronic healthcare records (EHR) from general practice may be a good source of data for pharmacoepidemiology studies. However, some conditions have significant periods of management in secondary care or specialist centres and electronic data from one source may not capture all the events happening in another setting. In order to answer a specific research question, researchers must choose which data source(s) are most appropriate in order to ensure the findings are generalisable and that case misclassification can be minimised.

The impact of using different populations in EHR studies has not been evaluated. A previous publication investigated mortality following venous thromboembolism (VTE) using the combination of primary care, secondary care hospital episode statistics (HES) and mortality data from the Office for National Statistics (ONS) from the Clinical Practice Research Datalink (CPRD) [1]. Many of the symptoms of VTE present in primary care and a high level of validity of VTE recording in primary care has been reported previously [2,3]. More serious cases may first appear in secondary care or as a cause of death in the death certificate, without any record in the primary or secondary care records. General practitioners may not obtain the full information on the cause of death; feedback from secondary care can be poor, therefore death data can be non-coded or missing. Considering the range of recording options, this condition is an ideal candidate to use as an example of when there is a need to use multiple data sources to examine the patient journey in full.

Previous research has highlighted that case validity is improved by using more than one source of data [4], minimising misclassification of either the exposure or outcome, or both. The objective of this study was to investigate the impact of the choice of data source in estimating mortality following VTE, with a secondary aim to investigate the influence of the denominator definition. In order to do this we recreated an analysis of mortality following VTE using CPRD primary care data and linked HES and ONS mortality data, using the same version of the data sources and same study period [5]. We experimented with using the primary care data only and including available linked data.

## Materials and Methods

**Fig 1:**
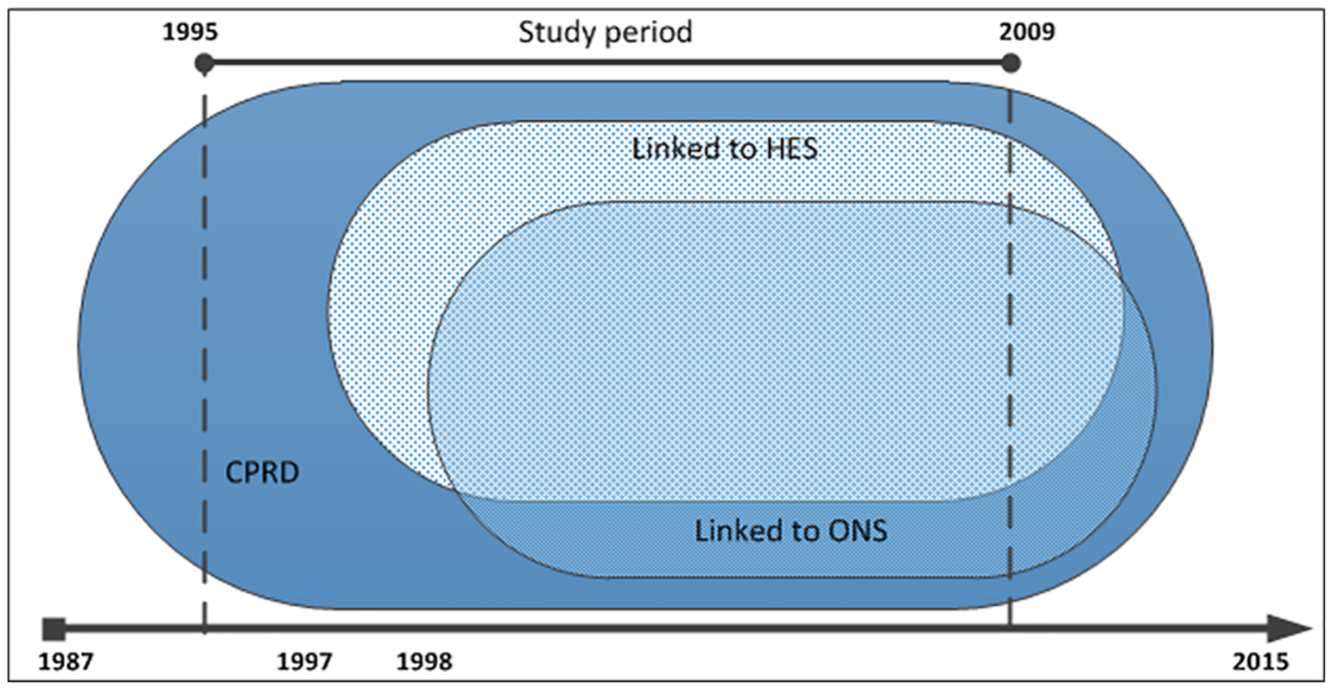
Availability of data within the study period.

The primary care data from CPRD is a database of computerised medical records from across UK General Practice. Data are available from 1987 on over 13.6 million patients [6] in a dynamic cohort where patients can join/leave a GP practice over the course of follow up. A systematic review of validation studies found that medical data in CPRD were generally of high validity [7]. The national Hospital Episodes Statistics (HES) data contain details of all admissions to National Health Service (NHS) hospitals in England from April 1997 [8]. The Office for National Statistics (ONS) mortality data contains the date and coded cause of death for the population of England and Wales from January 1998 [9]. HES and ONS mortality data are deterministically linked to the primary care data using a combination of identifiers including the patient's unique NHS number, gender, date of birth and postcode. For this study, each individual data source had a different period of coverage, the CPRD linkage was limited to consenting GP practices from England, and not all of the patients registered at a participating practice were eligible for each linkage (Fig 1).

**Fig 2:**
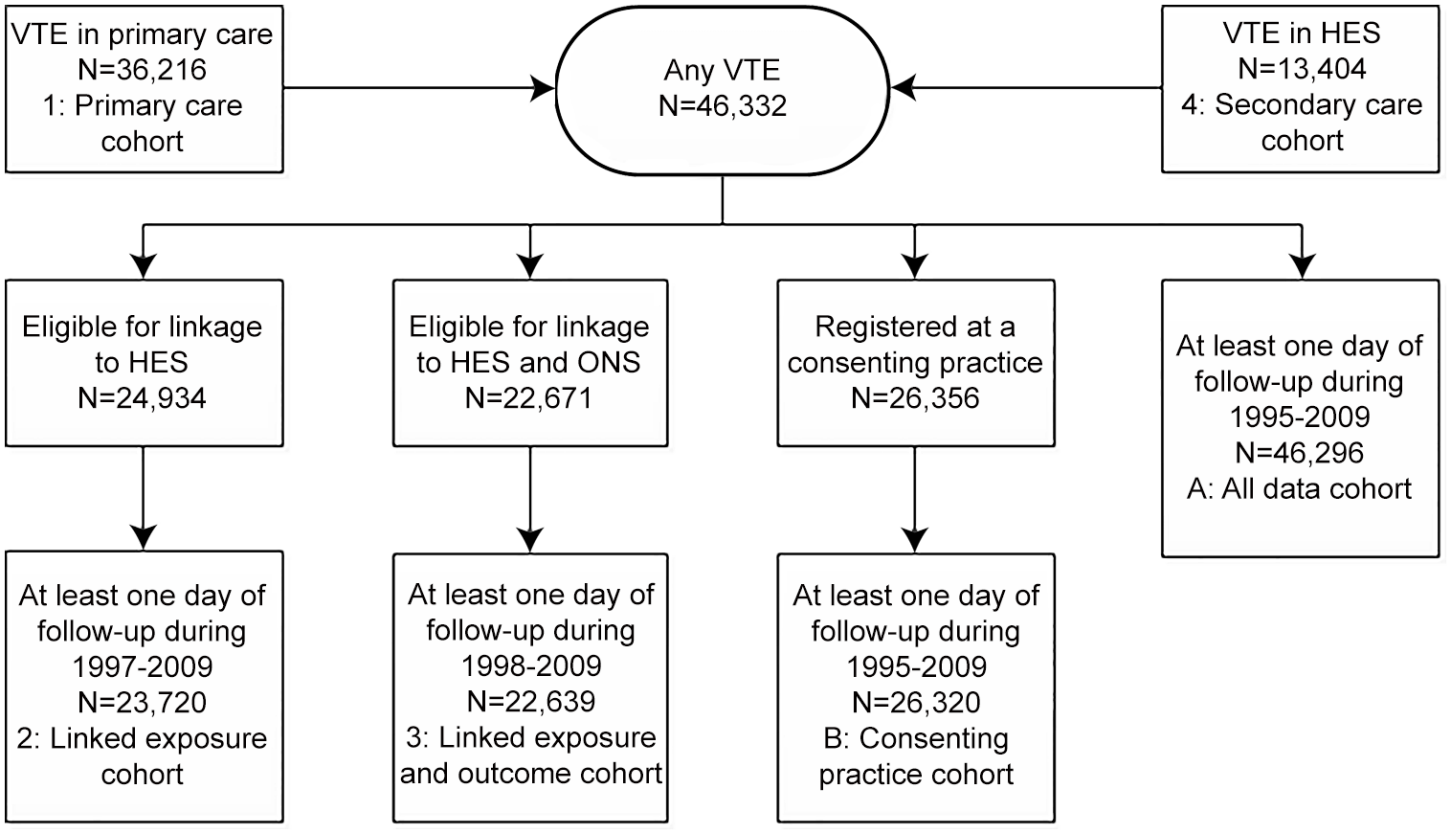
Flow chart of eligibility.

We recreated an analysis of mortality following VTE using the same study period and data sources as in a previous publication [5]. An older version of the linked data was used covering the period up to the 30th October 2009, at which point 40% of CPRD practices had consented to take part in the linkage. VTE was defined as a composite of distal and proximal deep venous thrombosis (DVT) and pulmonary embolism (PE) from either primary or secondary care. In order to imitate making different choices, multiple cohorts were identified with the data sources available (Fig 2). In all cohorts, patients were aged 18 years or older with a diagnosis of VTE (index date) on or after 01/01/1995.

Four cohorts were identified in order to assess the impact of the choice of data source:

1. Primary care cohort; data on exposure (VTE) and outcome (death) from primary care only, for the whole study period 1995–2009
2. Linked exposure cohort; including linked exposure data from HES, for eligible patients and the overlapping study period 1997–2009
3. Linked exposure and outcome cohort; including linked exposure data from HES and linked outcome data from ONS, for eligible patients and the overlapping the study period 1998–2009
4. Secondary care cohort; data on exposure from HES only and outcome data from primary care, for the overlapping study period 1997–2009

Patients were categorised by their prior VTE risk based on modifiable (fractures, surgery, trauma) or unmodifiable (cancer, congestive heart failure, varicose veins) factors recorded before the index date. Patients were followed from the index date until censoring, based upon the earliest of the end of data collection, patient's death, or transfer out of the practice, whichever date came first. The date of death was identified from the primary care data or linked ONS mortality data, where available. The mortality incidence rate for each of the cohorts was calculated per 1,000 person years (/1000py). Survival analyses using Cox proportional hazards regression was used to estimate the relative hazard rates (RRs) for all-cause mortality over time. RRs were calculated by gender and age using those aged 18–39 as the reference. The statistically adjusted model included age, gender, lifestyle information (BMI, smoking status, alcohol use) and VTE risk category.

Sensitivity analyses evaluated the influence of the denominator definition by considering individual eligibility and data coverage periods in two further cohorts; patients with VTE from either primary or secondary care over the whole study period (cohort A: all data cohort), and patients from consenting practices whether or not they were individually eligible (cohort B: consenting practices).

The CPRD Group has obtained ethical approval from a National Research Ethics Service Committee (NRES) for all purely observational research using anonymised CPRD data; namely, studies which do not include patient involvement (which is the vast majority of CPRD studies). Individual patient consent is not required for observational studies using anonymised CPRD data. Individual studies must be granted approval by the Independent Scientific Advisory Committee for MHRA database research (ISAC). This study was approved under protocol number 14/024.

## Results

A total of 46,332 patients were identified with a record of VTE from either primary or secondary care between 1995 and 2009. The largest cohort was of those diagnosed in primary care (cohort 1: N = 36,216, 71%), of which 3,320 (9%) were diagnosed before April 1997 when linked HES data became available and 6,539 (18%) were registered at practices in Wales, Scotland or Northern Ireland. Over 70% of patients had no obvious risk factor of VTE. In the HES data, 13,404 patients were identified with a diagnosis of VTE between 1997 and 2009 (cohort 4), of which 3,288 (25%) had a matching record in primary care on the same date. After pooling the primary care and HES patients and restricting to those eligible for HES linkage and to the overlapping coverage period (1997–2009), the linked exposure cohort included 23,720 cases (cohort 2). The average follow up period for cohort 2 was shorter than cohort 1 by 0.5 years (3.54 vs. 4.08 years) and patients were more likely to have a modifiable risk factor for VTE (28.7% vs. 21.3%). The linked exposure and outcome cohort was created by restricting to those eligible for ONS linkage and to the overlapping coverage period (1998–2009), including 22,639 cases (cohort 3). The average follow up period for cohort 3 was shorter than for both cohorts 1 and 2 at 3.39 years. The broadest cohort included all patients regardless of eligibility for linkage or whether the practice had consented (cohort A: all data cohort). This cohort included 46,296 patients of which 5,365 patients (11.6%) were diagnosed before mortality statistics were available from the ONS. The cohort of patients from consenting practices with a diagnosis in either primary care or HES included 26,320 patients (cohort B: consenting practices). A larger proportion of these were diagnosed in primary care (49% vs 43% in the other linked cohorts). Table 1 compares patient characteristics between all cohorts and shows that the age, gender, smoking and BMI profile was similar for all cohorts. The mean age was 55–56 years and 55% of patients were female. Over half of the patients were overweight or obese at the time of diagnosis and one fifth were smokers.

**Table 1:** Baseline information on VTE patients at diagnosis.

Characteristic	1: Primary care cohort N = 36,216	2: Linked exposure cohort N = 23,720	3: Linked exposure and outcome cohort N = 22,639	4: Secondary care cohort N = 13,404	A: All Data cohort N = 46,296	B: Consenting practice cohort N = 26,320
*Mean follow-up time (sd)*	4.08 (3.56)	3.54 (3.24)	3.39 (3.08)	3.18 (3.20)	3.85 (3.50)	3.79 (3.55)
*VTE diagnosis point*, *N (%)*						
Primary care	36,216 (100%)	10,316 (43.5%)	9,814 (43.3%)	-	32,906 (71.1%)	12,930 (49.1%)
Hospital	-	10,116 (42.6%)	9,668 (42.7%)	13,404 (100%)	10,101 (21.8%)	10,101 (38.4%)
Both	-	3,288 (13.9%)	3,157 (13.9%)	-	3,289 (7.1%)	3,289 (12.5%)
*Year of diagnosis*, *N (%)*						
1995	1,394 (3.8%)	-	-	-	1,394 (3.0%)	691 (2.6%)
1996	1,490 (4.1%)	-	-	-	1,490 (3.2%)	744 (2.8%)
1997	1,600 (4.4%)	757 (3.2%)	-	413 (3.1%)	1,920 (4.1%)	1,078 (4.1%)
1998–2009	31,732 (87.6%)	22,963 (96.8%)	22,639 (100%)	12,991 (96.9%)	41,492 (89.6%)	23,807 (90.5%)
*Age*						
Mean (sd)	54.8 (17.9)	56.5 (17.6)	56.5 (17.6)	58.0 (17.4)	55.6 (17.8)	56.2 (17.7)
18–39	3,451 (9.5%)	1,895 (8.0%)	1,815 (8.0%)	935 (7.0%)	4,127 (8.9%)	2,199 (8.4%)
40–59	9,231 (25.5%)	5,444 (23.0%)	5,197 (23.0%)	2,746 (20.5%)	11,196 (24.2%)	6,129 (23.3%)
60–79	16,515 (45.6%)	11,064 (46.6%)	10,536 (46.5%)	6,362 (47.5%)	21,266 (45.9%)	12,198 (46.3%)
80+	7,019 (19.4%)	5,317 (22.4%)	5,091 (22.5%)	3,361 (25.1%)	9,707 (21.0%)	5,794 (22.0%)
*Gender*, *N (%)*						
Women	19,974 (55.2%)	13,244 (55.8%)	12,620 (55.7%)	7,366 (55.0%)	25,559 (55.2%)	14,664 (55.7%)
Men	16,242 (44.8%)	10,476 (44.2%)	10,019 (44.3%)	6,038 (45.0%)	20,737 (44.8%)	11,656 (44.3%)
*BMI*, *N (%)*						
Underweight	1,630 (4.5%)	1,172 (4.9%)	1,132 (5.0%)	692 (5.2%)	2,179 (4.7%)	1,274 (4.8%)
Normal	8,539 (23.6%)	5,770 (24.3%)	5,523 (24.4%)	3,267 (24.4%)	11,032 (23.8%)	6,387 (24.3%)
Overweight	11,506 (31.8%)	7,380 (31.1%)	7,030 (31.1%)	4,082 (30.5%)	14,473 (31.3%)	8,203 (31.2%)
Obese	9,771 (27.0%)	6,185 (26.1%)	5,960 (26.3%)	3,288 (24.5%)	12,162 (26.3%)	6,741 (25.6%)
Unknown BMI	4,770 (13.2%)	3,213 (13.5%)	2,994 (13.2%)	2,075 (15.5%)	6,450 (13.9%)	3,715 (14.1%)
*Smoking Status*, *N (%)*						
Non Smoker	16,541 (45.7%)	10,667 (45.0%)	10,101 (44.6%)	5,928 (44.2%)	20,960 (45.3%)	11,958 (45.4%)
Ex-Smoker	9,187 (25.4%)	6,615 (27.9%)	6,468 (28.6%)	3,804 (28.4%)	12,024 (26.0%)	6,983 (26.5%)
Smoker	7,151 (19.7%)	4,412 (18.6%)	4,225 (18.7%)	2,440 (18.2%)	8,970 (19.4%)	4,953 (18.8%)
Unknown Smoking Status	3,337 (9.2%)	2,026 (8.5%)	1,845 (8.1%)	1,232 (9.2%)	4,342 (9.4%)	2,426 (9.2%)
*Alcohol Drinking Status*, *N (%)*						
Non Drinker	5,056 (14.0%)	3,010 (12.7%)	2,838 (12.5%)	1,717 (12.8%)	6,387 (13.8%)	3,405 (12.9%)
Ex-Drinker	2,045 (5.6%)	1,478 (6.2%)	1,447 (6.4%)	829 (6.2%)	2,672 (5.8%)	1,546 (5.9%)
Drinker	22,634 (62.5%)	15,157 (63.9%)	14,546 (64.3%)	8,451 (63.0%)	28,852 (62.3%)	16,659 (63.3%)
Unknown Drinking Status	6,481 (17.9%)	4,075 (17.2%)	3,808 (16.8%)	2,407 (18.0%)	8,385 (18.1%)	4,710 (17.9%)
*Risk category for VTE prior to index*, *N (%)*						
Modifiable strong^α^	2,923 (8.1%)	3,205 (13.5%)	3,067 (13.5%)	1,825 (13.6%)	4,292 (9.3%)	3,326 (12.6%)
Modifiable moderate/low^β^	4,792 (13.2%)	3,604 (15.2%)	3,424 (15.1%)	2,117 (15.8%)	6,442 (13.9%)	3,937 (15.0%)
Unmodifiable^γ^	2,231 (6.2%)	1,647 (6.9%)	1,570 (6.9%)	1,023 (7.6%)	3,064 (6.6%)	1,833 (7.0%)
No obvious risk factor	26,270 (72.5%)	15,264 (64.4%)	14,578 (64.4%)	8,439 (63.0%)	32,498 (70.2%)	17,224 (65.4%)
*Region*, *N (%)*						
North East	607 (1.7%)	276 (1.2%)	261 (1.2%)	171 (1.3%)	731 (1.6%)	308 (1.2%)
North West	5,280 (14.6%)	4,406 (18.6%)	4,157 (18.4%)	2,615 (19.5%)	7,376 (15.9%)	4,863 (18.5%)
Yorkshire & The Humber	1,953 (5.4%)	1,318 (5.6%)	1,266 (5.6%)	819 (6.1%)	2,600 (5.6%)	1,446 (5.5%)
East Midlands	1,999 (5.5%)	885 (3.7%)	848 (3.7%)	518 (3.9%)	2,393 (5.2%)	996 (3.8%)
West Midlands	3,471 (9.6%)	3,097 (13.1%)	2,965 (13.1%)	1,706 (12.7%)	4,753 (10.3%)	3,350 (12.7%)
East of England	3,668 (10.1%)	3,231 (13.6%)	3,047 (13.5%)	1,728 (12.9%)	4,980 (10.8%)	3,641 (13.8%)
South West	2,874 (7.9%)	3,245 (13.7%)	3,081 (13.6%)	1,976 (14.7%)	4,368 (9.4%)	3,613 (13.7%)
South Central	3,884 (10.7%)	2,636 (11.1%)	2,543 (11.2%)	1,462 (10.9%)	4,792 (10.4%)	2,908 (11.0%)
London	3,111 (8.6%)	2,177 (9.2%)	2,124 (9.4%)	1,062 (7.9%)	3,955 (8.5%)	2,425 (9.2%)
South East Coast	2,830 (7.8%)	2,449 (10.3%)	2,347 (10.4%)	1,347 (10.0%)	3,809 (8.2%)	2,770 (10.5%)
Northern Ireland, Scotland or Wales	6,539 (18.1%)	0 (0.0%)	0 (0.0%)	0 (0.0%)	6,539 (14.1%)	0 (0.0%)

^α^ Modifiable (transient) strong risk factor for VTE: hip or leg fracture, hip or knee replacement, major surgery (pelvis, lower leg, knee, feet, veins, arteries, spinal cord, and unspecified region), major trauma (head, neck, spinal cord, trunk, pelvis, lower leg, knee, feet, and unspecified region).

^β^ Modifiable (transient) moderate/low risk factor for VTE: major surgery (head, neck, trunk, and arms/hands), major trauma (arms/hands), pneumonia, chronic obstructive pulmonary disease, hormone therapy, or oral contraceptives.

^γ^ Unmodifiable risk factor for VTE: active cancer, congestive heart failure, varicose veins.

**Fig 3:**
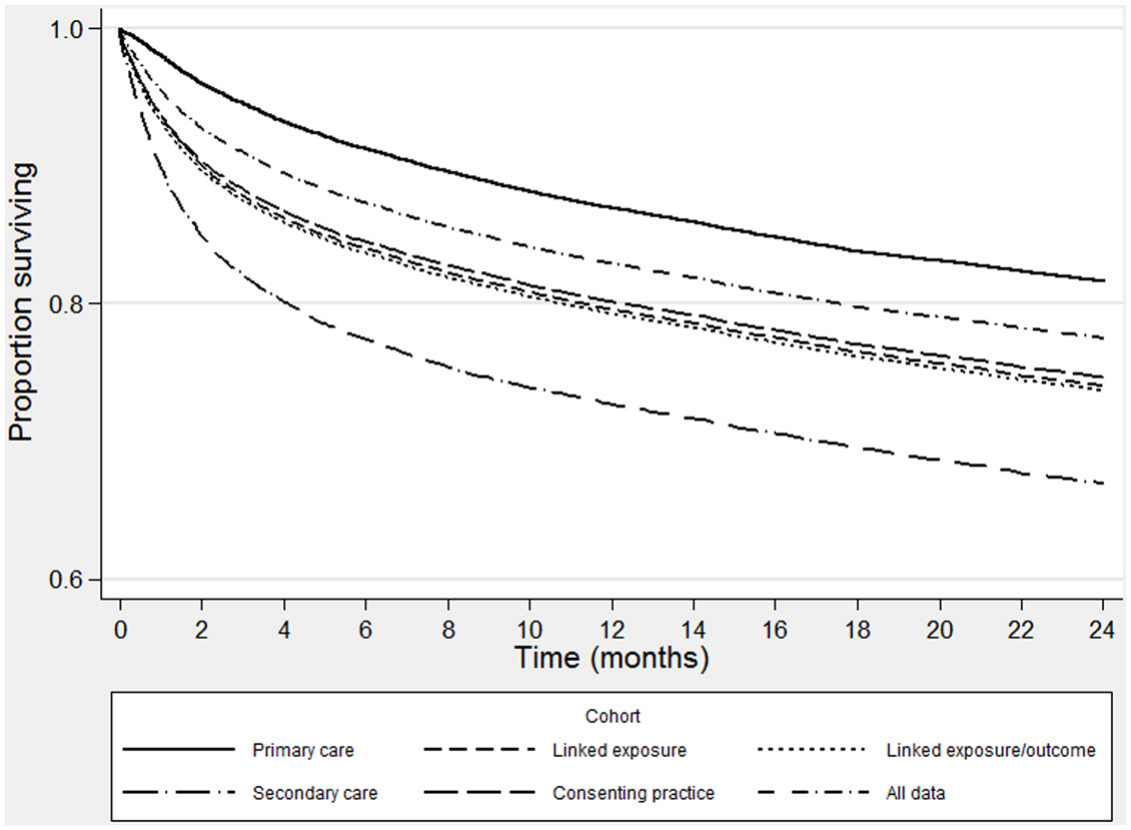
Mortality rate following VTE over time, by cohort.

Table 2 shows that the mortality rates over the 2 years following a VTE diagnosis were lowest in the primary care cohort (108.4/1000py) and highest for patients diagnosed in secondary care (237.2/1000py). Including eligible patients diagnosed in either setting (linked exposure cohort) produced a mortality rate between the two (169.9/1000py); adding mortality outcome data from ONS (linked exposure and outcome cohort) resulted in an increase in the mortality rate (173.2/1000py). Restricting to practices participating in the linkage, but ignoring individual eligibility resulted in a rate lower rate than the linked exposure and outcome cohort (164.3 vs 173.2/1000py). The largest number of deaths (9,701) was attributed to the all data cohort; however the mortality rate was the lowest of all the cohorts that included linked data (140.6/1000py). Fig 3 compares all cohorts over the two years.

**Table 2:** Mortality rates following VTE, by cohort.

Characteristic	N	Source of mortality data	Failures (deaths)	Person-time (years)	Incidence of Mortality per 1,000 person-years
1: Primary care cohort	36,216	primary care	6,112	56362	108.4
2: Linked exposure cohort	23,720	primary care	5,771	33961	169.9
3: Linked exposure and linked outcome cohort	22,639	primary care or ONS	5,569	32149	173.2
4: Secondary care cohort	13,404	primary care	4,186	17651	237.2
*A*: *All data cohort*	46,296	primary care or ONS	9,701	69001	140.6
*B*: *Consenting practice cohort*	26,320	primary care or ONS	6,264	38125	164.3

Table 3 shows that 85% of all fatal cases were in patients aged 60 or over. Older patients, especially the elderly, had an increased risk of mortality after adjusting for gender, lifestyle information (BMI, smoking status, alcohol use) and VTE risk category. A larger number of deaths were identified in women, however after adjustment, the mortality risk was found to be higher in men. The relative rate of mortality by gender and age were comparable across the cohorts although there was a lower mortality rate in the reference group for age (18–39) in the primary care cohort than all of the others.

**Table 3:** Relative hazard rates (RRs) for all-cause mortality following VTE, by cohort.

	1: Primary care cohort N = 36,216			2: Linked exposure cohort N = 23,720			3: Linked exposure and outcome cohort N = 22,639			4: Secondary care cohort N = 13,404			A: All data cohort N = 46,296			B: Consenting practice cohort N = 26,320		
Characteristic	N deaths	IR	Adjusted RR §	N deaths	IR	Adjusted RR §	N deaths	IR	Adjusted RR §	N deaths	IR	Adjusted RR §	N deaths	IR	Adjusted RR §	N deaths	IR	Adjusted RR §
*All*	6,112	108.4	-	5,771	169.9	-	5,569	173.2	-	4,186	237.2	-	9,701	140.6	-	6,264	164.3	-
*Age*																		
18–39	89	15.5	Reference	99	32.4	Reference	95	32.7	Reference	72	49.7	Reference	154	22.8	Reference	107	30.0	Reference
40–59	859	56.3	3.99 (3.21–4.97)**	710	81.2	2.67 (2.16–3.29)**	684	82.4	2.70 (2.17–3.34)**	502	119.2	2.54 (1.98–3.25)**	1,283	70.5	3.32 (2.81–3.93)**	801	81.1	2.87 (2.34–3.51)**
60–79	3,095	120.4	8.54 (6.91–10.56)**	2,837	177.2	5.63 (4.60–6.89)**	2,735	181	5.72 (4.65–7.03)**	2,036	237.4	4.75 (3.75–6.02)**	4,795	150.5	6.92 (5.89–8.13)**	3,065	171.7	5.88 (4.84–7.14)**
80+	2,069	213.9	12.19 (9.83–15.10)**	2,125	345.4	8.76 (7.14–10.74)**	2,055	352.1	8.87 (7.21–10.93)**	1,576	461.9	7.24 (5.70–9.20)**	3,469	285.1	10.45 (8.88–12.31)**	2,291	335.9	9.18 (7.54–11.16)**
*Gender*																		
Men	2,744	108.8		2,565	170.9	Reference	2,469	173.4	Reference	1,867	231.7	Reference	4,315	139.4		2,784	164.7	
Women	3,368	108.1	0.90 (0.85–0.95)**	3,206	169.2	0.88 (0.83–0.93)**	3,100	173.1	0.89 (0.84–0.94)**	2,319	241.8	0.92 (0.86–0.98)**	5,386	141.6	0.90 (0.87–0.94)**	3,480	164	0.88 (0.84–0.93)**

^§^ RR (95 CI), adjusted for age, gender, lifestyle information (BMI, smoking status, alcohol use) and VTE risk category

IR: Incidence rate per 1,000 person-years

** P<0.05

## Discussion

We looked at the impact of the choice of data source in estimating mortality following VTE and found considerable differences in the observed mortality rate across cohorts built using different sources, despite comparable relative rates by gender and age. All of the cohorts we examined were similar in terms of age, gender and lifestyle information; however the mortality rate was two times higher for patients diagnosed in secondary care data compared to primary care. Using linked data sources provided estimates between the two; however the choice of denominator in linked populations also affected the estimates reported, with lower rates seen when individual eligibility was not considered and the whole study period was used. Fig 3 shows the variation in rates across all of the cohorts over two years, highlighting that the difference can be seen immediately following diagnosis. This suggests that the cohorts were representing different populations.

We repeated a previously published study, modifying the study design to identify four cohorts that could feasibly be used to analyse mortality following VTE. We estimated the incidence rate for each cohort and the relative risk of mortality comparing those aged 18–39 to other age categories and comparing men to women. The primary care cohort represented the patient set and analyses that would be performed if only general practice records were available. Both the exposure (VTE) and outcome (death) were identified from primary care records and data were available for the full study period 1995–2009. The mortality rate (108.4/1000py) was comparable to a previously published study (97.8/1000py) [10], however there are potential limitations on the suitability of studying VTE in a primary care cohort and the availability and accuracy of the mortality information.

A pulmonary embolism is a potentially life-threatening condition with common symptoms including chest pains and a shortness of breath. Patients with such symptoms are more likely to attend the hospital and one third of patients with symptomatic VTE manifest pulmonary embolism [11]. In analysing only patients diagnosed in secondary care, we found a mortality rate of more than double that in the primary care cohort (237.2 vs. 108.4/1000py). The overlap between cohorts was relatively small (7%), suggesting they were very different patient populations. This study is in line with previous research highlighting how data sources do not always concur [12,13,14]. Movig et al (2002) compared clinical coded diagnoses from hospital with laboratory data and found a large number of patients (approximately 87%) identified via laboratory data did not have an associated coded diagnosis [14]. A study based on coded data may return a differing result to one based on laboratory results much like we have found when comparing patients diagnosed in primary versus secondary care.

Previous research has highlighted that case validity is improved by using a combination of linked data sources. Herrett et al (2013) used four linked data sources to analyse acute myocardial infarction and found that each individual data source missed a substantial proportion (25–50%) of MI events, suggesting that failure to used linked data is likely to lead to biased estimates [4]. A more representative cohort of VTE cases may therefore include exposures from both primary and secondary care. The linked exposure cohort included diagnoses from both data sources for the overlapping coverage period (1997–2009). The mortality rate (169.9/1000py) was halfway between what was seen in the primary care (108.4/1000py) and secondary care (237.2/1000py) cohorts. This may have addressed potential misclassification of the exposure, however misclassification of the outcome may persist through the use of primary care data for the outcome.

Although the gatekeepers to healthcare access in the UK, holding the longitudinal record for patients, the GP may not always be informed of a patient's death in good time, and in some cases not at all. Within the GP record, some patients may have multiple records relating to death on different dates without clarity on which is correct. The ONS mortality dataset includes information on the date and cause of death from civil registration records. All deaths in England and Wales have to be registered and usually within five days. With just one record per person and up-to date recording, this provides the gold-standard data source for mortality information which could reduce the level of misclassification of the outcome. The addition of ONS mortality data with the relevant coverage period (1998–2009), resulted in a smaller cohort (N = 22,639) with reduced person time (32,149) and an increased rate of 173.2/1000py. This cohort may have minimised misclassification, however it is not clear whether combining primary and secondary care data sources evaluates two different populations, where one comprising VTE cases presenting at hospital may be more severe.

Studies analysing linked data sources are frequently used in regulatory decision making, however previous publications have applied differing approaches in the definition of the denominator. With this in mind, we looked at the influence of not considering individual eligibility and data coverage periods. Linked data are not available for all patients in CPRD. Consent is given at the practice level and identifiers are sent to a trusted third party for linkage. Some registered patients may not be eligible for linkage if they have incomplete identifier information, withdraw consent or live outside of England. The consenting practice cohort included all patients from these practices regardless of eligibility. The cohort was necessarily larger than the linked cohorts, with more patients diagnosed in primary care (49%) and longer follow-up, presenting a lower mortality rate. The all data cohort explored eligibility further by including all patients and incorporating linked data where it was available; resembling the patient set and analyses reported in some previous publications [15,16,17]. This cohort primarily included patients from primary care (71%), and included longer follow-up, presenting a mortality rate 20% lower than the linked exposure and outcome cohort. This lower rate is not unexpected since 11.6% of the patients in this cohort were diagnosed before the start of coverage of the ONS data creating an immortal time bias. These sensitivity analyses highlight the importance of considering the eligibility in the methodology using linked data alongside the influence of the two different populations.

There were certain limitations to this study. We did not confirm the VTE record or validate the death information; further analyses of the cause of death could highlight that some of deaths were unrelated to VTE. In addition, cases fatal on index date and those where the VTE was diagnosed as part of a post-mortem may not have been included in the study. We did not have access to data from outpatients or emergency visits. The secondary care cohort is limited to those linked to the primary care record rather than all patients presenting to hospital with VTE. It is important to note that the data sources are collected for use in routine clinical practice, for audit purposes or for the purpose of reporting national statistics rather than for research. The linked population was limited to England only, however a previous comparison of those included in the linkage scheme to the whole CPRD population found no significant differences in patient characteristics [1] and similarly we found no substantial differences between the cohorts in this study (Table 1).

The size of the available cohort from CPRD makes the primary care EHR a viable choice for studying VTE however given the likelihood that more severe patients will present in secondary care, a linked population including patients presenting in secondary care may be a more appropriate source for analysis. Consideration of the influence of the denominator definition is important in studies of linked data as restricting the study population to those eligible for linkage does reduce the follow-up time, which is likely to have a large influence on small cohorts or those with rare outcomes. Future studies should consider the impact of the data source on case validity alongside the benefits of using linked data, together with the denominator definition. The choice of data source in this study demonstrated a substantial impact on the absolute rates of mortality following VTE. We used mortality following VTE as a learning case and expect these findings also may have an impact on other disease/outcome pairs.
